# Sandstone chemical consolidation and wettability improvement using furan polymer-based nanofluid

**DOI:** 10.1038/s41598-024-56007-3

**Published:** 2024-03-04

**Authors:** Matin Dargi, Ehsan Khamehchi, Fatemeh Ghallath

**Affiliations:** Department of Petroleum Engineering, Amirkabir University of Technology, Tehran, Iran

**Keywords:** Resin, Polymer, Chemical consolidation, Sand production, Nanofluid, Chemistry, Energy science and technology, Engineering, Nanoscience and technology

## Abstract

The oil and gas industry faces a challenge in meeting global energy demand due to sand production in unconsolidated or semi-consolidated reservoirs, leading to equipment wear, production instability, and significant financial burdens. Mechanical and chemical sand control methods are being used among which chemical sand consolidation techniques have emerged as a promising solution. In this research, furan polymer-based nanofluid is investigated as a chemical consolidant to explore its intriguing properties and characteristics and how the quantity of nanoparticles influences the fundamental properties of curing resin and wettability while pioneering a groundbreaking approach to enhancing regaining permeability. According to the findings, a substantial boost in core compressive strength has been achieved as well as an impressive increase in re-permeability, especially for the foam injection case, by the meticulous optimization of nanofluid composition. The results include a remarkable regain permeability of 91.37%, a robust compressive strength of 1812.05 psi, and a noteworthy 15.32-degree shift towards water-wet wettability. Furthermore, silica nanoparticles were incorporated to enhance the thermal stability of the fluid, rendering it more adaptable to higher temperatures. Therefore, Furan polymer-based nanofluid is not only expected to present a solution to the challenge of sand production in the oil and gas industry but also to provide operational sustainability.

## Introduction

As an inseparable part of the modern world's economy, the oil and gas industry needs to boost hydrocarbon production rate to meet our ever-growing energy demands^1^. However, when production rates exceed a certain threshold, a domino effect of issues can be set in motion, and one of the most significant concerns is sand production. This increase in fluid production rate is primarily achieved by reducing bottom-hole pressure, and as time goes on, it can lead to a range of problems including the challenging occurrence of two-phase flow, water coning, and condensate buildup near the wellbore. As a result, the pressure exerted on oil and gas reservoirs intensifies, giving rise to increased drag forces and in-situ stress, ultimately exacerbating the problem of sand production.

Sand production is defined as the transportation of sand particles from the reservoir to the wellbore through the flowing fluids during the hydrocarbon production process. This challenge is particularly pronounced in wells linked to unconsolidated or partially consolidated reservoir rocks, accounting for nearly 70% of the world's hydrocarbon reservoirs. An astonishing fluctuation in the concentration of sand particles in oil pipe systems has been reported; from a manageable 1.0% to 40.0% in which operational issues occur such as pipe and separator erosion, instability in production cavities, and complications in well control^2–6^. Dealing with sand production issues places a substantial financial burden on the oil and gas industry, with costs running into hundreds of millions of dollars each year. It's crucial to note that a significant portion of the world's hydrocarbons is located in poorly consolidated reservoirs, characterized by their geological youth and the absence of natural cementation between rock grains. This makes them exceptionally susceptible to sand production, especially when factors like changes in in-situ stress, high oil production rates, cavity collapses, and the presence of water in the formation come into play^7,8^.

Field observations have revealed that sand production is not a uniform challenge since its concentration varies from well to well. However, its consistent vexing consequences encompassing equipment erosion, safety hazards such as well-blowouts and fires may force us to halt production, leading to additional expenses and resource consumption through the drilling of more wells to achieve the same hydrocarbon recovery^9–11^.

To combat these sand-related challenges and maintain the efficiency of hydrocarbon production, the industry employs an array of sand control methods. These methods fall into two main categories: mechanical and chemical. Mechanical solutions involve the use of stand-alone screens, expandable sand screens, and slotted liner systems. On the other hand, chemical methods involve the application of sand consolidation techniques to prevent sand movement. One approach entails filling the formation with resin-coated particulate solids, while another wets the unconsolidated sand with a bonding resin. There's also a method that places resin-treated sand between the loose sand in the formation and the wellbore to create a screen. The varying degrees of success and associated costs of these techniques have also been added to the challenges faced by the oil industry. Furthermore, there are a variety of resin systems including epoxy, furan, furfuryl alcohol, phenol–formaldehyde, and urea–formaldehyde. The purpose of using all of them is to artificially enhance the cementation of the matrix of the reservoir rock and increase its compressive strength. A lot of work has been done on various resin systems and their benefits and drawbacks have been compared. The advantages of furan over other resin systems were reported as its corrosion resistance to the inorganic acid, high-temperature stability, economic viability, good wetting properties, curing when heated without adding the curing agent, miscibility with a myriad of thermosetting resins and forming a lot of products with diverse performance, and having the property to shrink certain parts of hydrated clay within the formation sand and enhancing the permeability of the consolidated mass^12–17^. While sand production issues continue to strain operations and budgets, the critical flow rate, which is the point at which sand is entrained with produced fluids, further complicates the issue. Besides, sand production is influenced by a complex interplay of factors, categorized into driving, resisting, and operational elements. Among unconsolidated sandstone reservoirs, those with permeability between 0.5 and 8 Darcie are most susceptible. The problem can manifest early in the life of a well or develop over time, influenced by variables like reservoir pressure, water breakthrough, and fluid velocities.

In an industry that plays a critical role in global energy supply, sand production remains a long-standing and formidable challenge. It impacts not only production rates but also the safety and sustainability of operations^3,11,12,18–20^. Chemical sand consolidation, introduced in the 1940s, has emerged as a promising technique, but it still has a long way to go before it becomes more accessible and cost-effective for a broader range of reservoirs.

In a study by Talaghat et al. (2009), a suitable resin for consolidating Asmari oil wells in Ahwaz and Mansoori oil fields was identified. Among six tested resins, only a phenol–formaldehyde resin exhibited the desired characteristics, including permeability, porosity, and compressive strength^5^. Lahalih and Ghloum (2010) tested polymeric compositions on Kuwait dune sands and Minagish oil well MN-117 sand, achieving compressive strengths of 35–100 kg/cm^2^, exceeding oil formation recommendations. These compositions effectively halted water production, with higher temperatures necessitating increased polymer doses^21^. Rickman (2012) explore laboratory experiments on ABR treatment fluids for consolidating weak formation sands and proppant packs. Foamed ABRs enhance proppant retention and permeability, offering solutions for sand control and proppant-flowback control in weak formations^22^. The study by Andrew and Dulu (2013) assesses sand consolidating resins for Niger Delta oil fields. Epoxy A&B outperforms other resins, with Furan following closely, while local resins, including Rubber Latex, show lower effectiveness in maintaining permeability and porosity under different pressures^23^. Mishra and Ojha (2016) created a chemical mixture of organic resins and inorganic chemicals to solidify loose sand, achieving a high uniaxial compressive strength of 1300 psi while maintaining porosity and permeability. Optimized parameters reduced chemical usage^24^. In 2016, Mishra and Ojha also introduced a novel consolidating agent using nano-SiO_2_ in urea formaldehyde to improve sand grain binding, enhance compressive strength, and water resistance. Their study analyzed interactions with FT-IR and TGA, explored clay's impact on consolidation, and assessed pressure drawdown and flow rate effects, showing potential for enhancing hydrocarbon production and flow assurance in field applications^25^. Jin Liu et al. (2018) examined the impact of organic polymer on soil stabilization through unconfined compression, direct shear, and tensile tests. They observed enhanced strength as polymer concentrations increased, as polymer membranes formed stable sand particle connections, though effectiveness depended on sand density^26^. Marandi et al. (2018) developed polymeric hydrogels to enhance sand consolidation in southern Iranian sandstone oil reservoirs. Their optimized hydrogel reduced sand production by 90% and modified water and oil permeabilities, providing a dual solution for sand control and permeability adjustment in field operations^27^. In Sugihardjo's (2020) study, a laboratory experiment assessed a sand consolidation chemical's ability to enhance rock grain bonding without major permeability loss. They analyzed reservoir rock and fluid properties, conducted core flooding experiments with synthetic and native cores. The synthetic cores reduced permeability initially, while cutting core ends improved it. Native cores had a significant permeability decrease. Suggestions for future improvements include increasing the injected oil rate during curing^28^. Tabbakhzadeh et al. (2020) studied chemical effects on epoxy and furan sand systems' properties. Models predict parameters with a 10% error, aiding oil and gas industry consolidation operations^29^. In the study by Alanqari et al. (2019), they examined a resin formulation, initially used at temperatures below 225°F, to determine its suitability for use in temperatures exceeding 290°F. The study explored a new formulation, employed amine curing, and conducted polymer analysis^30^. Nejati et al. (2023) present a novel epoxy-based nanofluid, Epoxy/g–C_3_N_4_–NS, for sand control in oil wells. It reinforces epoxy with carbon nitride nanosheets, employs a bubbling agent to enhance permeability, and substantiates its efficacy through analyses and experiments, highlighting its potential as a commercial sand control solution for oil wells^31^.

This research has been conducted chiefly to explore the potential of furan resin as a chemical stabilizing agent. A key innovation of this study is the employment of the exceptional properties of silica nanoparticles and the utilization of the SDS surfactant to enhance the stabilizer’s efficacy. The study is designed to optimize the fluid composition to achieve maximal compressive strength, increased re-permeability, and improved wettability of reservoir rocks. This involves adjusting the fluid’s formula in the static phase, followed by the injection of the optimized fluid, in both non-foamy and foamy forms, into outcrop samples during the dynamic phase.

## Methodology and materials

### Materials

Furan nanofluid consisting of furan resin (CF306), hardener (H002), isopropyl alcohol, silica nanoparticles and SDS (Sodium Dodecyl Sulfate) surfactant, was used in this study. According to the SARA analysis and ICP-MS of the oil and water of Ahwaz field formation, the oil with 29.55 API contains 58% saturated compounds, 6% resin, 3.2% asphaltene, 20.1% aromatics, and the formation water has Na, K, Ca and Mg ions with the highest values of 59,800, 1184, 7531.4 and 1085.9 ppm, respectively.

**Figure 1:**
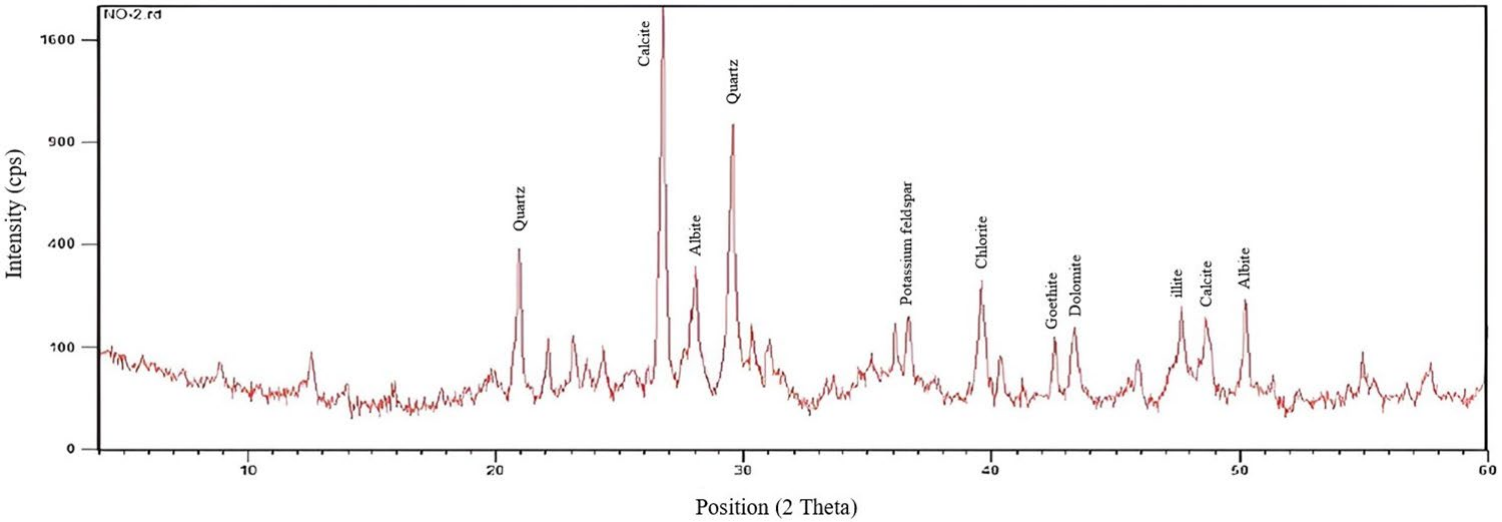
The result of the XRD test of the outcrop sample of the Asmari layer.

To mimic the reservoir conditions as closely as possible, the Asmari formation outcrop was used, and the graph obtained from the XRD analysis is shown in Fig. 1.

The analysis indicates that quartz and calcite are the predominant minerals constituting 29% and 31% of the rock’s composition, respectively. Additionally, the clay minerals chlorite and illite are present, comprising 9 and 7% of the structure. The outcrop also contains other components, including dolomite at 4%, albite at 14%, potassium feldspar at 3%, and goethite at a minor 2%.

### Experimental

#### Procedure

**Figure 2:**
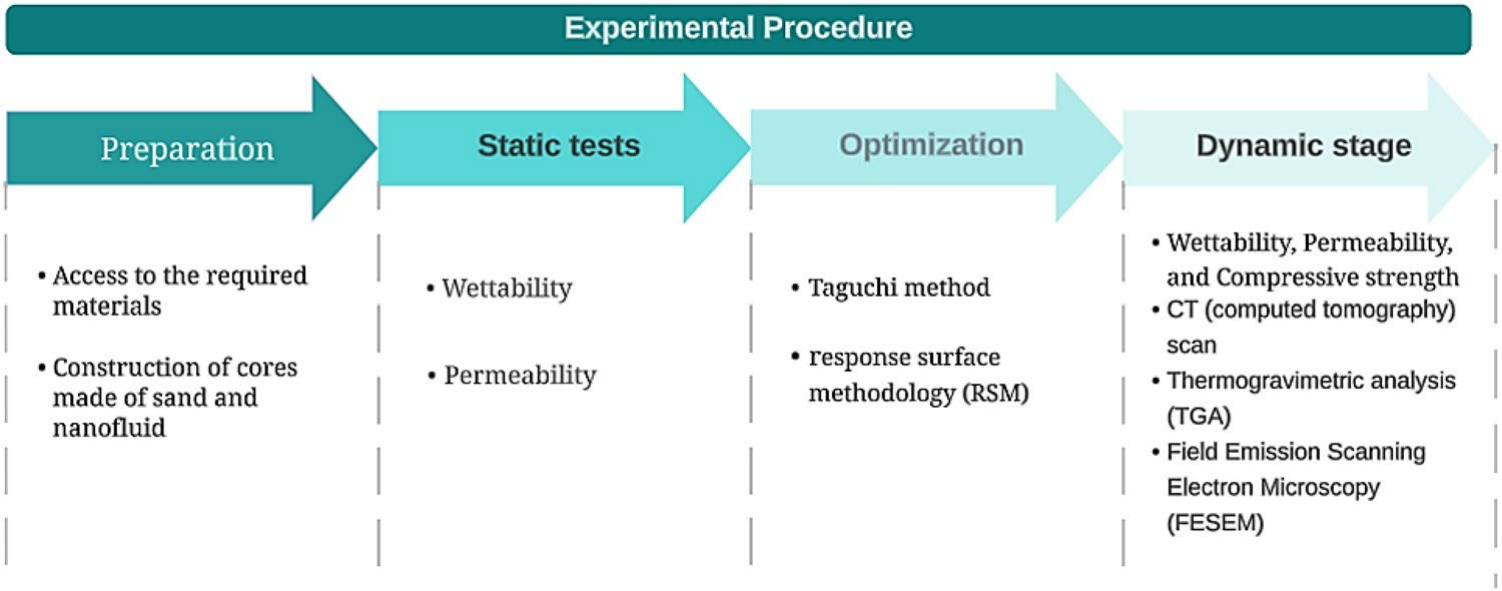
Workflow chart.

In this research, the Taguchi method is used to design experiments and determine the optimal composition, including solid content, hardener percentage, and nanoparticles. This approach was chosen to ensure reliable results while minimizing the number of experiments, thereby saving time and financial resources. The experiments entailed creating samples comprising a mixture of sand sourced from the outcrop and nanofluid and analyzing the permeability and wettability factors. After performing static tests and conducting optimization using Minitab software, the chosen fluid transitioned to the dynamic phase for core flood testing in which it is injected into the outcrop cores under real reservoir conditions. The optimal fluid was injected in two forms: non-foamy and foamy, in order to assess and compare their performance in returning permeability. The workflow is shown in Fig. 2.

#### Taguchi method and response surface methodology (RSM)

Design of Experiments (DoE) emerged as a valuable tool in the late twentieth century for enhancing product quality, both in Western countries and Japan. DoE employs statistical test design techniques to improve the development of new and existing products. Given the resource-intensive nature of testing, efficient experimentation is vital, especially when dealing with a growing number of variables, which can lead to significant cost and time increases. DoE is essential for obtaining comprehensive insights into processes, drawing logical conclusions, and documenting evidence while minimizing expenses and time.

The Taguchi method, a specific approach within DoE, focuses on improving processes and products by emphasizing efficient experimentation with minimal resource allocation. It considerably reduces the number of trials and experiments needed to gather crucial information by using sets of tables known as orthogonal arrays. These arrays enable the examination of primary and interaction effects with minimal experimentation. Various Taguchi orthogonal array models, such as L8 (representing 8 experiments), L16 (16 experiments), and L18 (18 experiments), among others, exist. The choice of array depends on the factors and levels selected for a particular investigation, as some may be more suitable while others impractical due to increased testing requirements^32,33^.

In this series of experiments, nano silica was used to improve chemical consolidation fluid performance. The amount of added nanoparticles can significantly influence changes in cured resin properties, such as compressive strength and core properties (permeability and wettability). Investigating the nanofluid performance with varying nanoparticle quantities was a primary objective. Quantities exceeding 1% by weight were not feasible due to challenges in nanoparticle dispersion in the fluid, and most nanoparticle applications are in weight percentages below 1%. Nano percentage was investigated at three levels: 0, 0.5, and 1, with zero indicating no nanoparticle use. Solids content was added in three levels: 10, 20, and 30, as exceeding 30% was detrimental to tank permeability, and below 10% did not result in effective curing. Similarly, the hardener was used at three levels: 10, 15, and 20, as less than 10% did not facilitate proper curing, and over 20% led to curing speed-related issues.

According to the aforementioned parameters and levels, 27 different conditions were initially considered, but to avoid extensive testing and substantial costs, the conditions were condensed into an L9 orthogonal array (comprising 9 tests) following Taguchi's method. Further details regarding this test design using Minitab software can be found in Table 1.

**Table 1:** The design was done by the Taguchi method.

Sample code	HC%	SC%	NC%
Aut-RF-1	10	10	**0**
Aut-RF-2	10	20	0.5
Aut-RF-3	10	30	1
Aut-RF-4	15	10	0.5
Aut-RF-5	15	20	1
Aut-RF-6	15	30	0
Aut-RF-7	20	10	1
Aut-RF-8	20	20	0
Aut-RF-9	20	30	0.5

The factors used in making these samples are defined as follows Eq. (1), (2) and (3).

1 $$SC\left(\%\right)= \frac{Weight\, of\,  Furan\, (g)}{Weight\, of\, (Furan+solvent)}\times 100$$

2 $$HC\left(\%\right)= \frac{Weight \,o\,f Hardener\, (g)}{Weight\, of\, Furan\, (g)}\times 100$$

3 $$NC\left(\%\right)= \frac{Weight\, of \,Nanoparticle\, (g)}{Weight\, of \,Solution\, (g)}\times 100$$

The RSM (Response Surface Methodology) is a mathematical and statistical approach for modeling and analyzing complex problems. It finds application when the desired outcome of the problem, known as the goal, is influenced by multiple independent variables, and the objective is to enhance this outcome. This method assesses the interplay between one or more responses by analyzing the impact of independent variables. Given the non-linear nature of the problem at hand, the RSM method leverages a quadratic model to optimize the composition of nanofluid components. In this research, RSM optimization based on the results obtained in the initial stage (utilizing the Taguchi method) has been employed^34^.

#### Preparation of samples

Nanofluid compositions with specific properties were prepared using sand sourced from outcrops in nine distinct states identified through the Taguchi's method. Water and oil had been absorbed by this sand from the formation, with an 80 to 20% ratio. It is worth noting that a UHP-400 ultrasonic homogenizer was employed to disperse nanoparticles within the fluid. The selection of a 20% fluid-to-sand ratio is based on the fact that the cores obtained from the outcrop have approximately 20% porosity in which the fluid is placed exclusively. The mixed sand and resin were combined and poured into cylindrical rubber molds. Subsequently, they were covered with foil and subjected to an oven at a temperature of 90 degrees Celsius.

### Static and dynamic stage tests

#### Permeability, wettability and compressive strength

The control of reservoir production rates strongly depends on the permeability of the reservoir rock which indicates the rock capacity to allow fluid to flow through its pores and channels^35,36^. Determining the extent of permeability reduction due to the infiltration of chemical fluids in the vicinity of the wellhead is of paramount importance in chemical consolidation procedures, as this determination aids in optimizing the chemical composition of the consolidation agent which is essential for controlling sand production, limiting permeability reduction, and minimizing formation damage. In this research, a gas permeability meter from the Fetco Company was utilized to measure the permeability of outcrop cores, sand, and resin samples. The samples ith higher permeability values that maintain open pores effectively, will then be selected. This parameter plays a major role in the optimization of the consolidant composition for the injection phase (dynamic testing). Wettability refers to the inclination of a fluid to disperse and adhere to a solid surface. Typically, the surface of sandstone rock formations tends to be water-wet, while carbonates tend to be oil-wet^37^. In chemical consolidation operations, wettability alteration may be induced as a result of chemical reactions and ion exchange between the rock and consolidation fluid^38^. Therefore, where the infiltration of chemical fluids can cause formation damage and permeability reduction, monitoring changes in wettability is necessary, as shifting toward water-wettability in the formation rock can enhance the relative permeability of water, increase oil production, and partially compensate the damage caused by the chemical fluid curing in the pores.

The specific setup employed to measure the contact angle in this research was made up of a light source, a small syringe, a beaker containing brine, a high-quality camera, and a computer equipped with Digimizer image analysis software. While each of the thin sections had been placed in the beaker in contact with the brine from the bottom, an oil drop was placed on the contact area using the small syringe in atmospheric conditions. The angle created by the denser fluid (brine) is considered the contact angle between the rock and the oil droplet. The image taken using the high-quality camera was imported into the Digimizer software to measure the contact angle and determine the wettability of the reservoir rock.

Before conducting field injection tests, it is essential to factor in various tests and considerations, including assessments of the resistance introduced by the polymer fluid. To achieve this, laboratory tests should be conducted to determine the compressive strength before and after fluid injection. These tests help establish whether the injected fluid effectively enhances resistance within the core sample. Since uniaxial compressive strength measurement is a destructive and damaging procedure for the core samples, an outcrop core, sharing similarities in terms of porosity and permeability with the injection cores was selected. The measured compressive strength of this chosen core is considered representative of the initial compressive strength of the injection cores.

#### Injecting nanofluid in liquid and foamy forms

To examine how core sample permeability, compressive strength, and wettability are affected by the injection of a polymer-based fluid, the properties of the outer cores were subjected to fluid injections and subsequent measurements. The objective was to improve the properties of the injected polymer-based fluid to enhance post-injection residual permeability and significantly increase compressive strength. Following various studies and investigations, promising results were obtained by using furan nanofluid in the form of foam, along with the addition of a surfactant.

**Figure 3:**
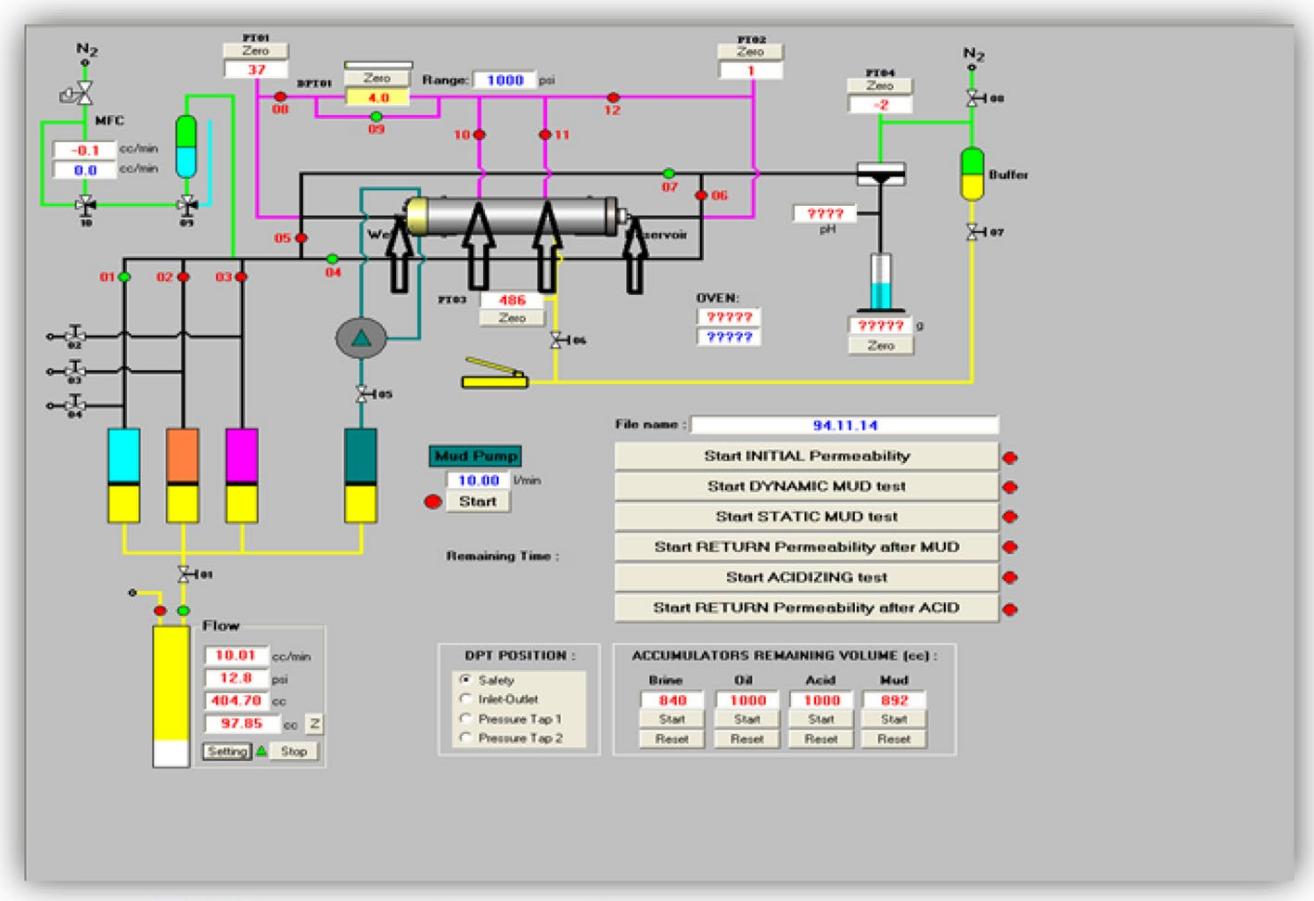
Schematic of formation damage device FDS350.

Two forms of furan polymer nanofluid injection, simple and foam, were employed in the cores, and permeability, compressive strength, and contact angles were measured. These properties were subsequently measured again after curing. Prior to the fluid injection using the FDS350 formation damage device, they were saturated with formation fluids. To prepare the cores for resin injection, diesel was employed as a pre-washing fluid to displace the formation fluid from the core pores and facilitate the conditions required for resin injection. Figure 3 shows the schematic of FDS350. In the foam injection case, 1% by weight SDS surfactant was used to foam furan nanofluid. After adding silica nanoparticles to the fluid and subjecting it to ultrasonic homogenization, SDS surfactant was introduced to the solution, and foaming was achieved using the Hamilton Beach HMD400 drink mixer. It is important to note that pre-wash fluid injection was performed at a volume equivalent to 2 pore volumes, while polymer-based fluid injection was conducted at 1 pore volume. Following fluid injection into the core and extraction from the opposite end, injection continued for some time to ensure fluid movement and distribution within the core. Subsequently, fluid injection ceased, and the core sample was placed within an autoclave operating at a temperature of 90 °C and a pressure of 120 bar to initiate the curing process of the polymer-based nanofluid sample within the core.

## Result and discussion

**Figure 4:**
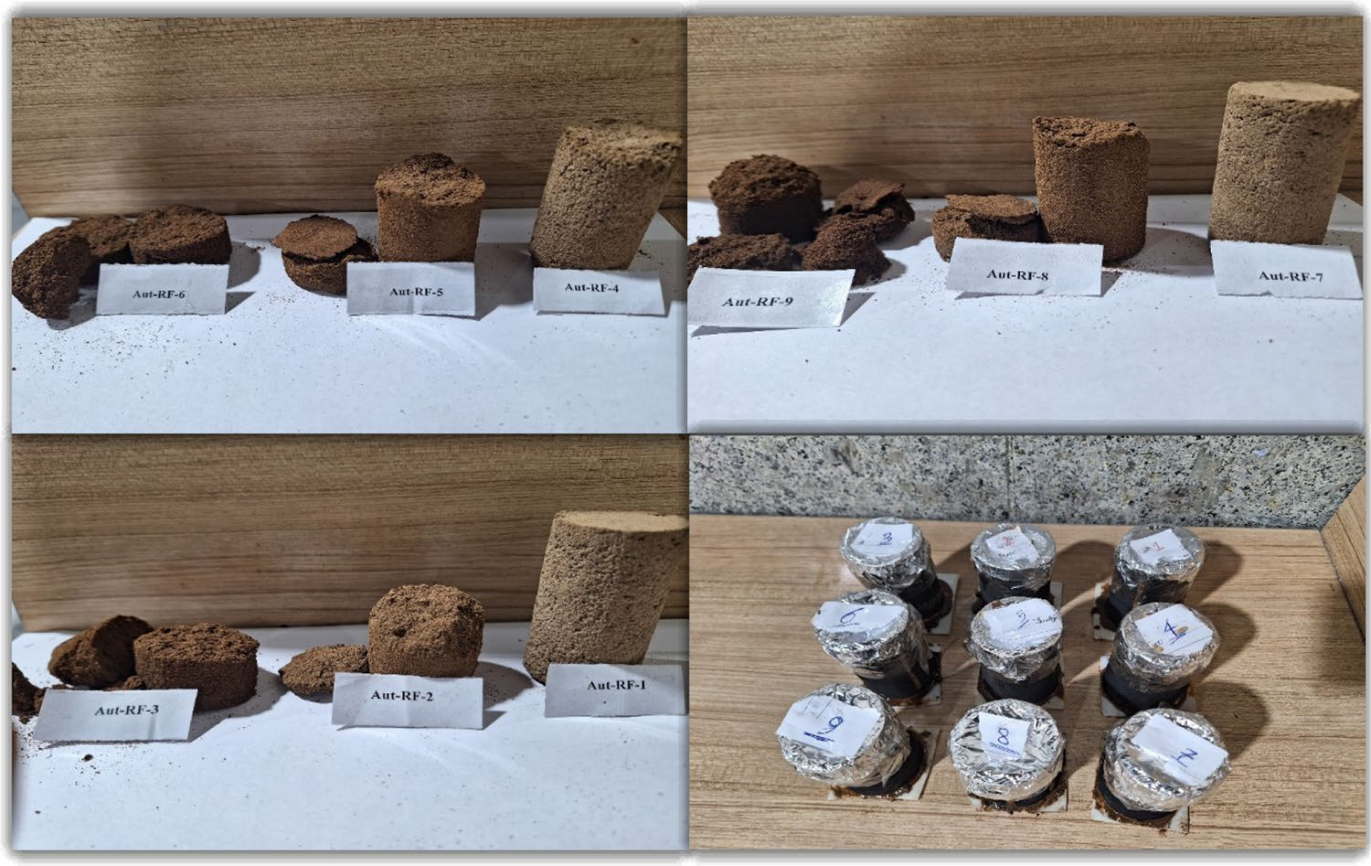
Cured samples (mixture of sand and polymeric nanofluid).

After 24 h, the samples were removed from the oven. The initial observations showed that only 3 of the 9 samples had relatively good strength and the rest were not completely hardened and had a paste state. While removing the samples from the rubber molds, samples code Aut-RF 1, 4 and 7 were removed safely, and the rest fell due to insufficient strength, leaving only pieces of them. In Fig. 4, the samples are shown after curing. The curing process means the transformation of the consolidation fluid from a liquid state to a solid state where polymer chains are formed between the consolidation fluid and the sands and the sand particles stick together. Although the other 6 samples also reached an outstanding strength after 24 h of exposure to air, due to the lack of air in the reservoir, they cannot be considered a suitable option for entering the next stage (dynamic stage).

### Static evaluation

#### Permeability and wettability

To measure wettability, the thin sections prepared from all three samples were individually immersed in formation water, with a drop of oil placed beneath them. The point where the oil drop made contact with the surface was captured in photographs, and the contact angle was then quantified using Digimizer software, as depicted in Fig. 8A–C. Based on the measured results, sample 4 exhibited the most water-friendly characteristics with an angle of 74.55, which was lower than the other two states. Conversely, sample 1 was identified as the most oil-friendly state, with an angle of 75.53.

The low compressive strength of all three samples (below 7 psi) didn’t allow us to precisely measure their exact values with the available equipment. Consequently, the impact of compressive strength was assessed in dynamic tests. Table 2 provides a brief summary of the static test results. To verify reproducibility, all nine cases were recreated, yielding similar outcomes. Only three samples with 10% solid content displayed satisfactory results, and their static test results are also presented in Table 2.

**Table 2:** The results of the static tests of the nanofluid.

Sample code	Contact angle (°)	Permeability (mD)
Aut-RF-1	76.51	248
Aut-RF-4	74.55	292
Aut-RF-7	75.84	260
Aut-RF-1N	77.2	210
Aut-RF-4N	73.41	310
Aut-RF-7N	75.54	294

#### Optimization by RSM

Since fluid injection into the core is extremely time-consuming and expensive, the optimal conditions for dynamic testing were determined using the aforementioned static tests results as well as Minitab software as a tool for applying the response surface methodology. To determine optimal configuration, all settings and their related results were entered and the goal was set to achieve the highest value of permeability and the lowest value of contact angle. This target was chosen because it represents the superior performance of the consolidation fluid, leading to the highest return permeability and greater hydrophilicity.

**Figure 5:**
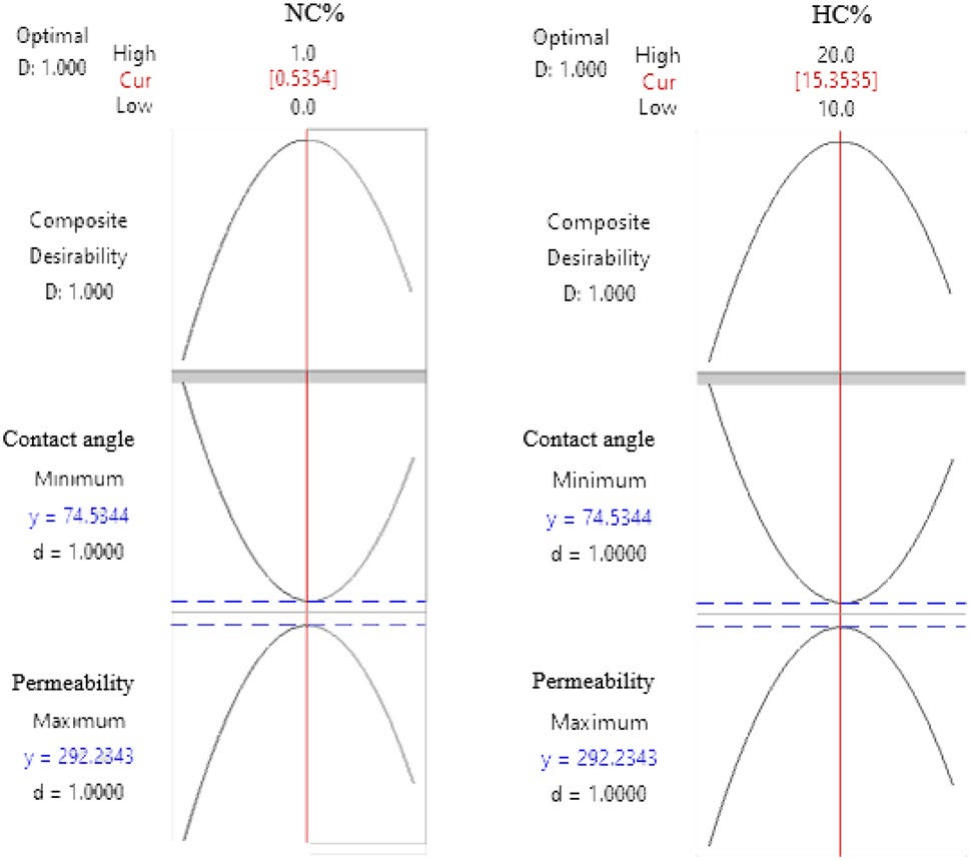
Response surface optimization diagram.

The results of the optimization are shown in Fig. 5. The graph shows that the changing trend of the contact angle related to the percentage of nanoparticles and the percentage of hardener is initially downward and then upward, so the lowest point is the bottom of the parabola. On the contrary, the permeability change trend related to the percentage of nanoparticles and the percentage of hardener first increases and then decreases, with the highest point being at the top of the parabola. As a result, in evaluating the combination's desirability, the optimal condition is determined as the middle point of the concentration range of nanoparticles and hardener.

### Dynamic evaluation

#### Effect of surfactant and nanoparticles

Dynamic experiments were conducted using outcrop cores to investigate the effect of polymer-based nanofluid injection on core permeability, compressive strength, and wettability. The aim was to increase the residual permeability after liquid injection, guided by the findings of previous research. The chosen approach involves the use of a nanofluid combined with SDS surfactant. Selected for its effectiveness in occupying pore spaces. The presence of SDS surfactant in the polymer fluid, when mixed in the process, reduces the viscosity and ensures better dispersion of the fluid in the porous medium. This resulted in more uniform curing in the medium and prevented clogging of the pores and throat openings. In addition, the surfactant's ability to reduce surface tension reduced capillary pressure and facilitated the movement of oil trapped in the core. Traditional methods of oil droplet removal were challenging and polymer nanofluid became a suitable alternative.

Previous studies by Khamehchi et al. (2016 and 2019) showed the effectiveness of SDS surfactant in creating microbubbles in Aphron liquid. Through analysis, an optimal weight percentage of 1% was identified as the best performance^39,40^. In the experiments, 1% by weight of SDS surfactant was added to the solution and thoroughly mixed using a high-speed mixer.

Notably, previous research often shows superior permeability reduction results with surfactant-assisted injection compared to simple injection^22,41^. After the injection of furan nanofluid into the cores with specific characteristics of permeability, compressive strength, and contact angle and the baking process, the properties of the cores were measured again.

**Figure 6:**
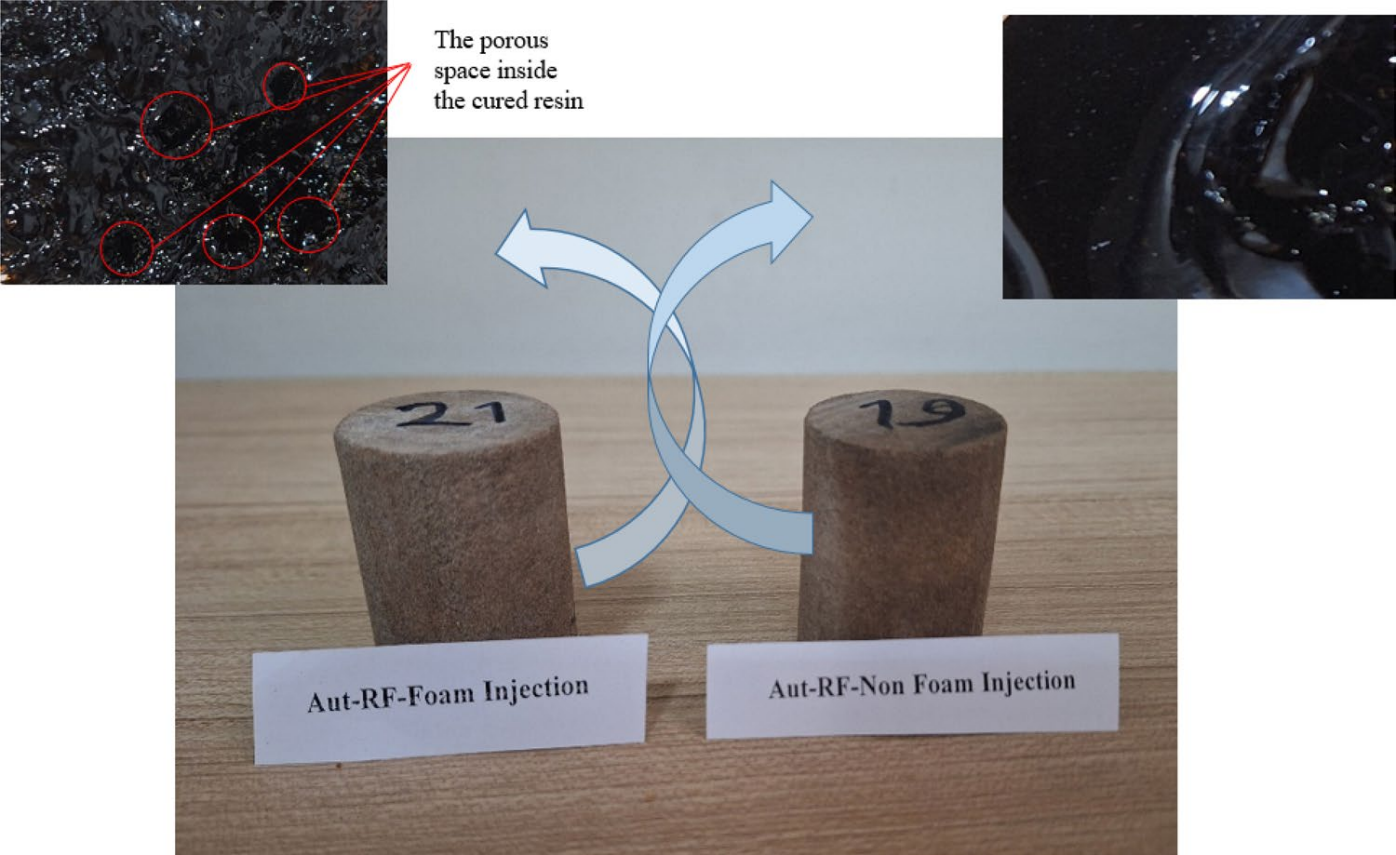
Samples of injected core after curing.

Consequently, the optimal nanofluid composition for core injection comprises 10% solid content, 0.53% nanoparticles, and 15.35% hardener. This composition is introduced into the core in both liquid and foam states. Figure 6 provides a visual representation of the core sample following the curing process. As depicted in the figure, the cured foam resin exhibits a porous structure, a feature absent in the cured liquid resin. One contributing factor to the superior performance of the foam state over the liquid state could be attributed to the presence of this porous structure, which prevents complete pore closure.

#### Wettability, permeability, and compressive strength

Following the curing of the samples, thin sections were extracted from the cores to assess wettability. The measurements revealed that the injection of furan polymer-based nanofluid, in both liquid and foam forms, significantly reduced the contact angle. In other words, it enhanced the water-wettability of both cores.

When a chemical consolidation agent is employed to solidify the formation, it could potentially lead to an undesired shift in sandstone wettability toward an oil-wet state. This issue could be particularly problematic in an oil reservoir. Consequently, assessing the influence of chemical consolidation agents is crucial, not only in terms of porosity and permeability reduction but also regarding wettability alteration.

**Figure 7:**
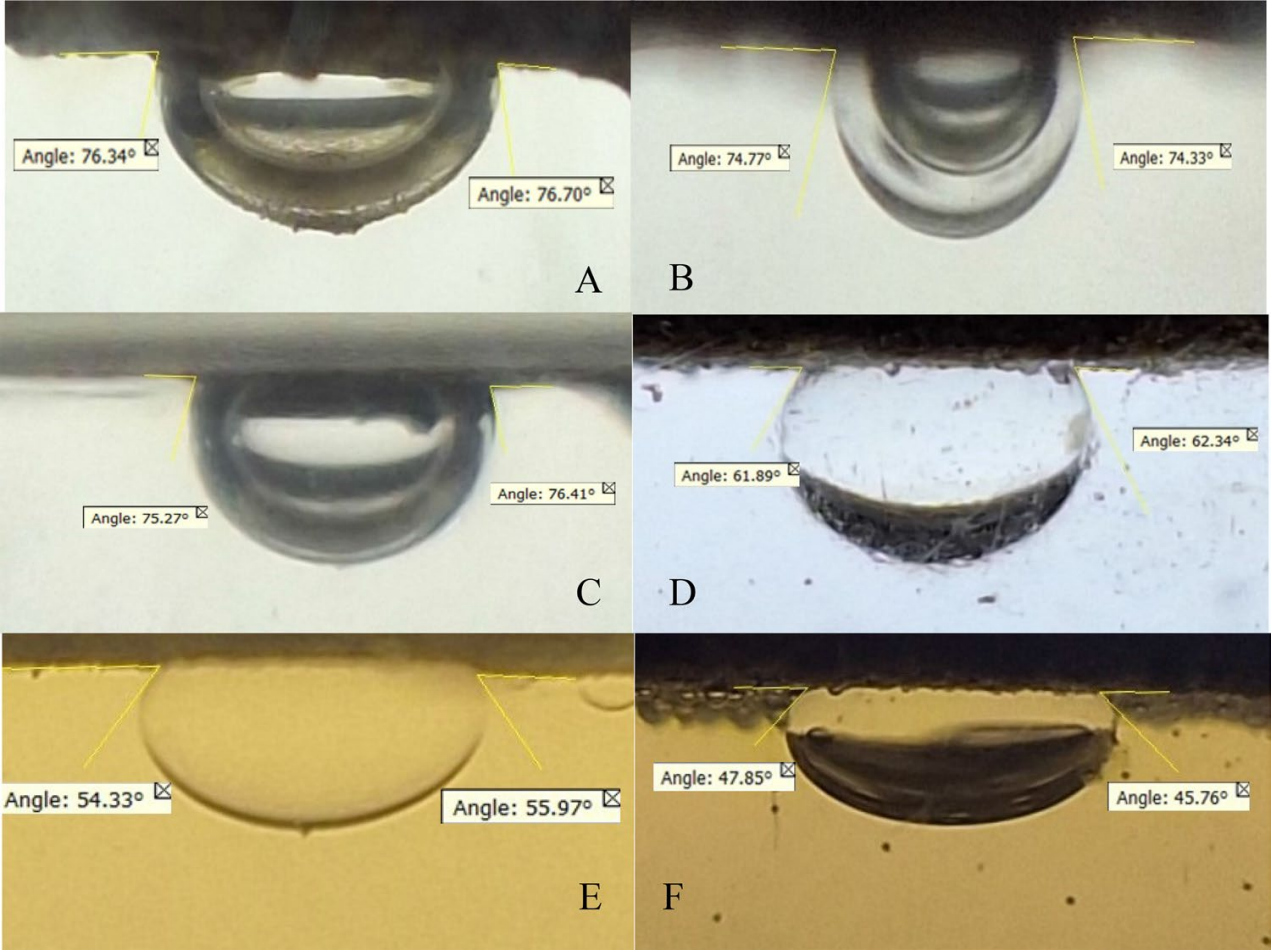
The contact angle of an oil drop (kerosene) and a thin section surface in the brine environment, in static (**A**–**C**), original (**D**), and dynamic state (**E**,**F**) (after liquid and foam injection).

In the case of simple injection, the contact angle decreased from 62.12 to 55.1, while surfactant-assisted injection lowered it even further to 46.8. These findings emphasize the superior performance of Furan polymer-based nanofluid with a surfactant in altering the wettability of reservoir rock to a more water-wet state. This outcome is of paramount importance as it can partially offset the reduction in permeability and the damage incurred by the reservoir due to consolidation fluid ingress through increased relative permeability. Figure 7 presents the contact angle values for the cores before injection (6D) and provides images of the contact angle after nanofluid injection in both simple (6E) and surfactant-assisted forms (6F).

**Figure 8:**
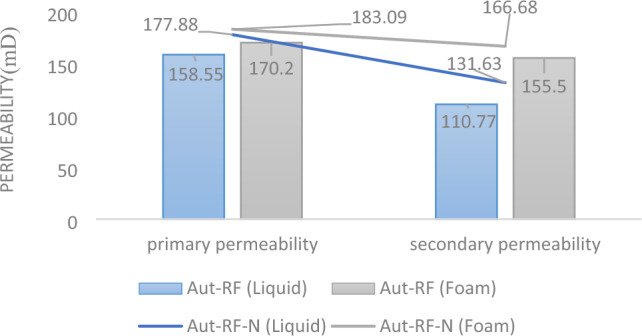
Permeability alterations within the core are depicted for two conditions: liquid and foam (Aut-RF), as well as for samples employed for reproducibility (Aut-RF-N).

**Figure 9:**
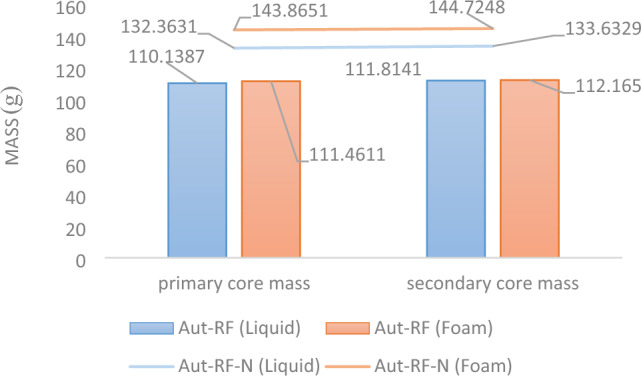
The mass parameter illustrates changes in the core for both the liquid and foam states (Aut-RF) along with samples employed for reproducibility (Aut-RF-N).

The assessment of permeability values post-injection and curing underscores the superior performance of surfactant-assisted injection in evaluating this parameter. In the case of simple injection, core permeability experienced a reduction of approximately 30%. However, with surfactant-assisted injection, this reduction was notably lower, at only 8.63%. Figure 8 presents the measured values for reference. In nanofluid injection with surfactant, the presence of air within the solution results in a lower resin content per unit volume compared to simple injection. This phenomenon is supported by the measured mass values of the core before and after injection in both liquid and foam forms, as depicted in Fig. 9.

**Figure 10:**
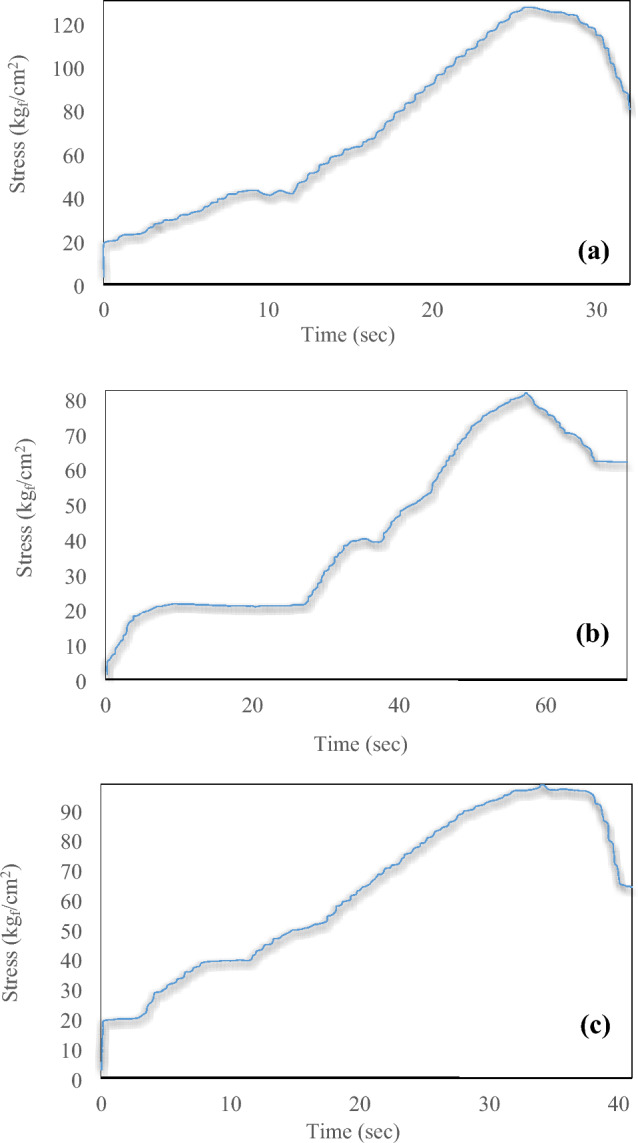
The stress-time diagram obtained from the UCS test of three cores: (**a**) after foam injection, (**b**) original, and (**c**) after liquid injection.

**Figure 11:**
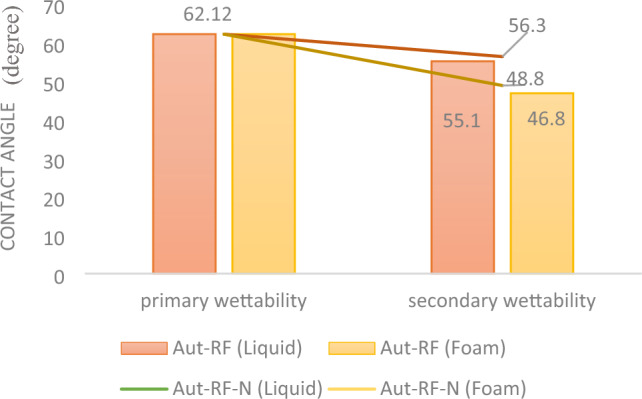
The wettability characteristic showcases variations in the core for both the liquid and foam states (Aut-RF), as well as for samples utilized to assess reproducibility (Aut-RF-N).

**Figure 12:**
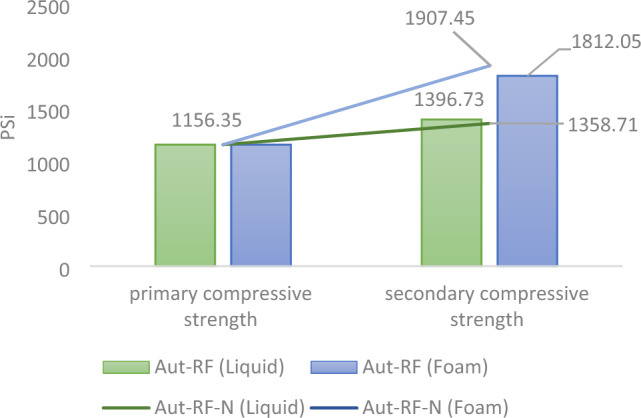
Compressive strength variations within the core are illustrated for both the liquid and foam states (Aut-RF), as well as for samples used for reproducibility (Aut-RF-N).

The initial compressive strength of the outcrop core, as the representative of the injection cores initial strength, measures 1156.35 psi. The compressive strength measurements for cores 1 (liquid) and 2 (foam) yielded values of 1396.73 psi and 1812.05 psi, respectively. The stress-time plot serves as a crucial instrument for assessing rock strength. These plots visually depict how applied stress relates to the duration of its application, providing valuable insights into how materials behave under varying conditions, as shown for the three cores in Fig. 10. The increase in core 2's (foam) strength by 655.7 psi, which surpasses core 1's (liquid) strength by 415.32 psi, can be attributed to the effective dispersion of nanofluid within the core. Notably, this improvement was achieved despite using a lower quantity of resin compared to core 1 (simple). Figure 11 and 12 provide a summary of the results from post-injection tests, showcasing the alterations in core characteristics. To enhance result reliability and verify repeatability, the aforementioned tests were also conducted with two new cores, with their results duly recorded.

#### CT (computed tomography) scan

**Figure 13:**
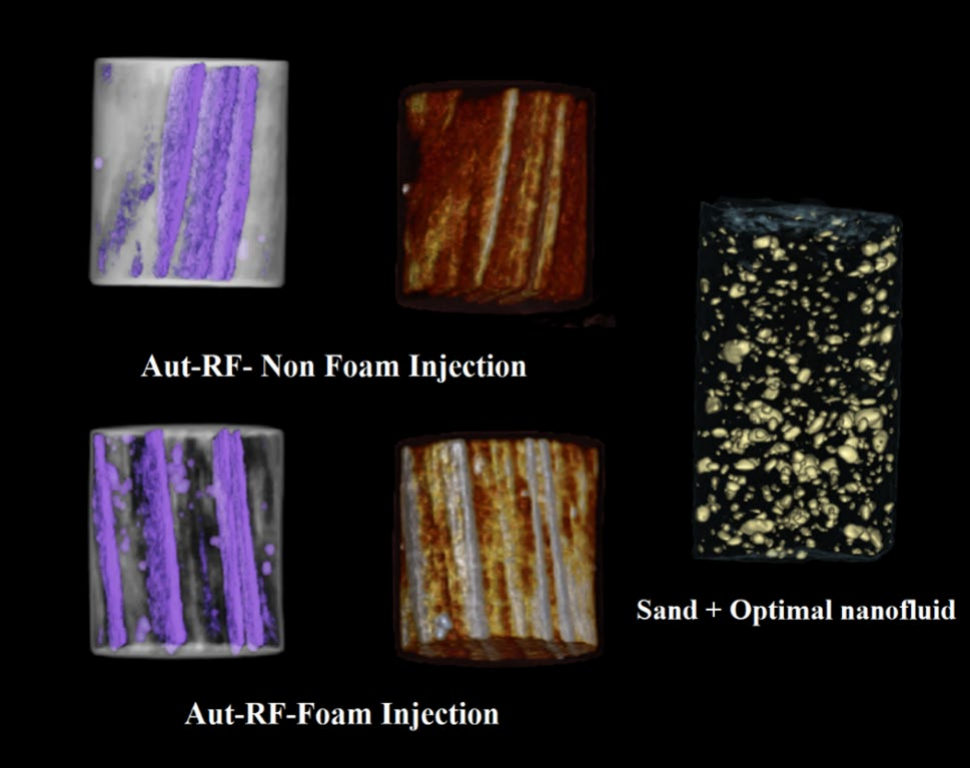
CT scan images of the injected core (liquid and foam) and cured samples (optimized sand and nanofluid).

Figure 13 visually demonstrates the placement of the injected nanofluid within the cores. The injected fluid forms a layer within the inner structure of the cores in both simple and surfactant cases. The key differentiator influencing core characteristics such as compressive strength and permeability post-injection is the arrangement of these layers. In simple injection, these layers are closely positioned to each other, resulting in limited localized strength. Conversely, in surfactant-assisted injection, the layers are effectively spaced apart, contributing to the development of requisite strength throughout the core. This accounts for the approximately 400 psi higher compressive strength observed in core 2 compared to core 1. The reduced permeability observed in the simple injection state can be attributed to the inadequate dispersion of the fluid, which predominantly follows the main and direct pathways. However, in both cases, despite injection into the core's center, the fluid, due to its lower viscosity and surface tension, traverses various paths, including pores, ensuring thorough dispersion.

In the sample containing optimal composition, nanofluid particles are widely distributed throughout the sample and function similarly to cement, binding the sand grains together. Notably, the cured nanofluid particles of furan are visible in golden color in Fig. 13.

#### Thermo-gravimetric analysis (TGA)

**Figure 14:**
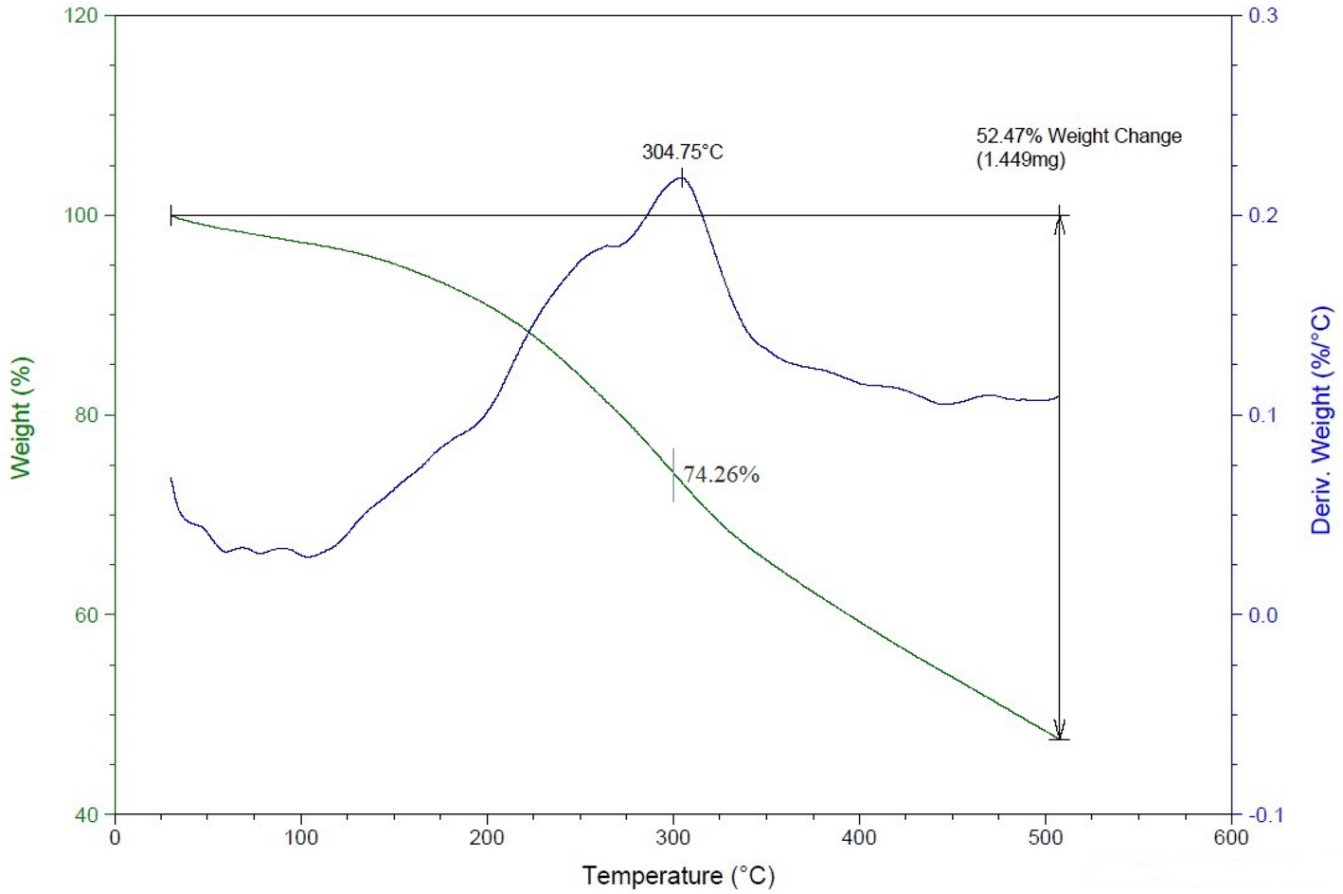
Thermal degradation diagram of the optimal fluid of the eruption containing nano-silica.

**Figure 15:**
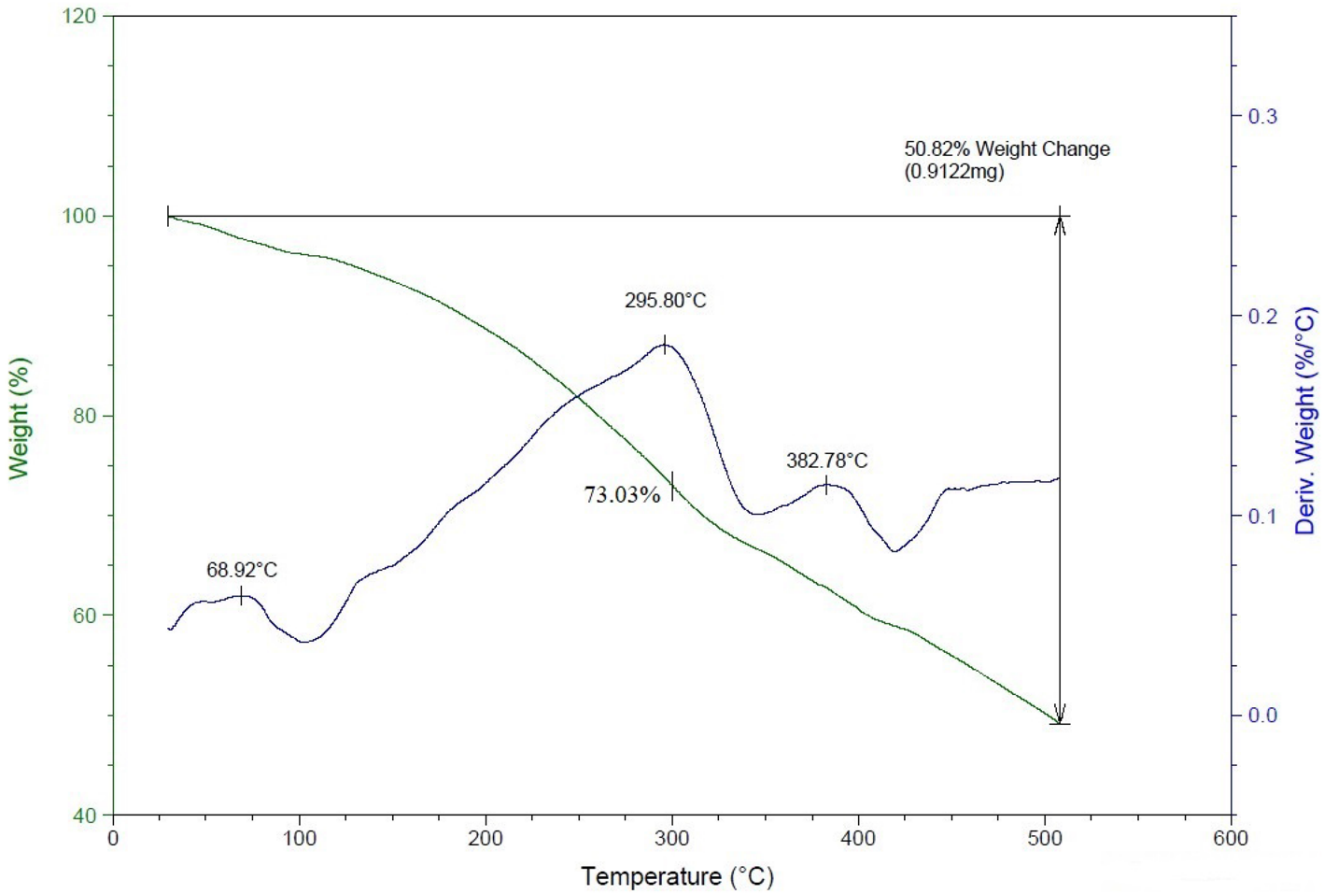
Thermal degradation diagram of the optimal fluid of the eruption without nano-silica.

To assess the impact of nanoparticles on the thermal stability and thermal degradation behavior of furan nanofluid, a relevant test was conducted for two distinct conditions: one without nanoparticles and the other with nanoparticles incorporated. Both sets of samples underwent a TGA test, ranging from ambient temperature to 500 °C, with a heating rate of 10°C/min within an air environment. The outcomes of these two scenarios are depicted in Figs. 14 and 15.

A comparison of these graphs reveals that, up to 300 °C, the sample lacking nanoparticles experiences greater degradation, with a remaining mass percentage of 73.03%. This underscores the beneficial effect of introducing silica nanoparticles to enhance the thermal stability of the sample.

For the sample devoid of nano-silica, maximum degradation occurs at approximately 295.8 °C. However, with the inclusion of nano-silica, this degradation temperature shifts to higher values, around 304.75 °C. The introduction of SiO_2_ has notably enhanced the material's thermal stability, evident from the comparison of two charts at an actual working temperature of 90 degrees Celsius. At this temperature, the chart containing nanoparticles displayed 97.61% mass remaining, surpassing the sample lacking silica nanoparticles by 1.03%. This exemplifies the beneficial impact of the nanoparticle on thermal stability at the operational temperature.

#### Field emission scanning electron microscopy (FESEM)

The FESEM images of the nanofluid within the core sample reveal that the resin is situated on the surface of the particles, with curing occurring in the same vicinity. This curing process of the resin on the particle surfaces promotes the bonding of sand particles, thereby augmenting the inter-grain strength within the core sample.

**Figure 16:**
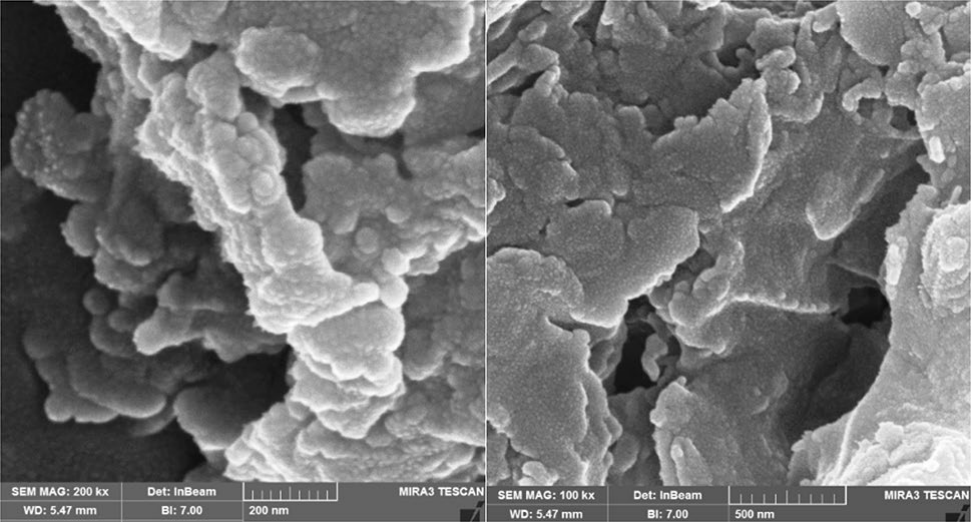
FESEM analysis related to the consolidated core sample.

Furthermore, vacant spaces between the sand particles are discernible, and the constriction points within the core sample structure continue to provide essential space for fluid mobility. Figure 16 prominently displays the presence of these constriction points. Additionally, the captured image vividly illustrates the cohesion of sand grains, as they adhere to each other.

### Contrast with prior investigations

To evaluate the effectiveness of the nanofluid discussed in this article, a comparative analysis was conducted between this nanofluid and other fluids that have been introduced in the previous studies related to the sandstone chemical consolidation (Table 3). Only H. Nejati et al. conducted studies on consolidation fluid that explored the impact of the injection fluid on altering wettability, leading to a decrease in the wettability of rock. What sets this research apart from other studies in this domain is the achievement of a 15.32 degree reduction in the contact angle, resulting in enhanced wettability of the reservoir rock. Furthermore, the introduced fluid demonstrated the most favorable performance in terms of regaining permeability.

**Table 3:** A brief literature review on sandstone consolidants in comparison with this study.

Literature	Fluid type	Regain permeability (%)	Compressive strength (psi)	Wettability variation
Dees (1992)^42^	An epoxy resin and a gas generator	50	7000	–
Fader et al. (1992)^43^	A modified furan resin	90	550	–
Parlar et al. (1998)^44^	Furan resin	80–85	2000	–
Cobianco et al. (1999)^45^	Sodium silicate solution	20–30	284–427	–
Larsen et al. (2006)^46^	Quasi Natural Consolidation (QNC) Ca^2+^, urease and urea	75	1531	–
Chaloupka et al. (2010)^47^	A catalyzed epoxy	63	–	–
Mishra and Ojha (2016)^24^	Urea–formaldehyde, potassium silicate and Ammonium chloride	64.65	1300	–
Mishra and Ojha (2016)^25^	Addition of nano-SiO_2_ in the urea–formaldehyde	88.44	2180	–
Al-Mulhem (2019)^48^	An epoxy resin and curing agent	70	–	–
H. Nejati et al. (2023)^31^	Epoxy/g–C_3_N_4_–NS nanofluid	86		The contact angle transitioned from 0 degrees to 24.4 degrees, signifying a shift toward reduced water wettability
This study	Furan nano fluid with SDS surfactant	91.37	1812.05	The contact angle shifted from 62.12 to 46.8, signifying increased water wettability

## Conclusion

Sand production continuously poses a production challenge in sandstone reservoirs. Addressing this issue involves using a chemical consolidation approach. This paper presents the use of furan polymer-based nanofluid for this purpose. The findings of the research and laboratory tests are stated below.

- A significant challenge in the performance of the nanofluid became apparent in the light of performing the static tests. A discernible lack of strength was observed within the amalgamation of sand and fluid when the solid content (SC) exceeded 10%. Consequently, an optimal fluid solution was achieved through meticulous optimization guided by the Response Surface Method. This solution incorporates 10% SC, 15.35% HC, and 0.53% NC, offering a more robust and effective outcome.
- The percentage of regained permeability in the outcrop sample exposed to foam injection, 91.37%, significantly surpassed the 70% achieved through liquid injection. Furthermore, the foam injection method substantially increases core compressive strength, attributed to the fluid placement within the pore space. The fluid forms well-dispersed layers in this injection modality, which markedly enhances performance.
- The superior performance of furan polymer-based nanofluid in combination with a surfactant can be introduced as a milestone in this research area. Furan polymer-based nanofluid injection into the sandstone reservoirs is an innovative solution to tackle the permeability reduction and formation damage caused by consolidation fluid ingress and assures the petroleum industry to enhance relative permeability greatly through wettability alteration of the rock towards a more water-wet state.
- The results of the thermal stability tests clearly illustrate the advantages of incorporating silica nanoparticles into Furan polymer-based fluid. The sample containing nanoparticles exhibits notably enhanced thermal stability, with a substantial shift in its degradation temperature to around 304.75°C. This observation highlights the positive impact of silica nanoparticles on the fluid's ability to withstand higher temperatures.

## Data Availability

All data generated or analysed during this study are included in this published article.

## References

[1] Khamehchi, E., et al., *Pipe Diameter Optimization and Two-Phase Flow Pressure Drop in Seabed Pipelines: A Genetic Algorithm Approach.*

[2] Tabar MA (2021). A comprehensive research in chemical consolidator/stabilizer agents on sand production control. J. Petrol. Explor. Prod. Technol..

[3] Ranjith P (2013). Effective parameters for sand production in unconsolidated formations: An experimental study. J. Petrol. Sci. Eng..

[4] Khamehchi E, Reisi E (2015). Sand production prediction using ratio of shear modulus to bulk compressibility (case study). Egypt. J. Pet..

[5] Talaghat M, Esmaeilzadeh F, Mowla D (2009). Sand production control by chemical consolidation. J. Petrol. Sci. Eng..

[6] Feng C (2012). Wettability modification of rock cores by fluorinated copolymer emulsion for the enhancement of gas and oil recovery. Appl. Surf. Sci..

[7] Abass H, Nasr-El-Din H, BaTaweel M, Abass H (2002). Sand control: Sand characterization, failure mechanisms, and completion methods. SPE Annual Technical Conference and Exhibition?.

[8] Araujo E, Araujo E (2014). Analytical prediction model of sand production integrating geomechanics for open hole and cased–perforated wells. SPE Heavy and Extra Heavy Oil Conference: Latin America.

[9] Tippie D, Kohlhaas C, Tippie D, Kohlhaas C (1973). Effect of flow rate on stability of unconsolidated producing sands. SPE Annual Technical Conference and Exhibition?.

[10] Rahmati H (2013). Review of sand production prediction models. J. Petrol. Eng..

[11] Safaei A (2023). Chemical Treatment for sand Production Control: A Review of materials, Methods, and Field Operations.

[12] Mishra S, Ojha K (2015). Chemical sand consolidation: An overview. J. Pet. Eng. Technol..

[13] Keith CI, Keith CI (2013). Coil tubing furan resin sand consolidation treatment on multi layered formation in Peninsular Malaysia. SPE Asia Pacific Oil and Gas Conference and Exhibition.

[14] Poston S, Poston S (1986). Development of a two-stage sand consolidation technique. SPE International Conference and Exhibition on Formation Damage Control.

[15] Alakbari FS (2020). Chemical sand consolidation: From polymers to nanoparticles. Polymers.

[16] Murphey J, Bila V, Totty K, Murphey J, Bila V, Totty K (1974). Sand consolidation systems placed with water. SPE Annual Technical Conference and Exhibition?.

[17] Marfo SA, Marfo SA (2015). Sand consolidation operations, challenges and remedy. SPE Nigeria Annual International Conrence and Exhibition.

[18] Dees JM (1993). Method of Sand Consolidation with Resin.

[19] Haggerty DJ, Haggerty DJ (2009). Sand consolidation testing in an API RP 19B Section IV perforation flow laboratory. 8th European Formation Damage Conference.

[20] Yeow L-M, Yeow L-M (2004). Sand production prediction study using empirical and laboratory approach for a multi-field gas development. SPE Asia Pacific Conference on Integrated Modelling for Asset Management.

[21] Lahalih S, Ghloum E, Lahalih S, Ghloum E (2010). Polymer compositions for sand consolidation in oil wells. SPE Production and Operations Conference and Exhibition.

[22] Nguyen PD, Rickman RD, Nguyen PD, Rickman RD (2012). Foaming aqueous-based curable treatment fluids enhances placement and consolidation performance. SPE International Conference and Exhibition on Formation Damage Control.

[23] Abanum AM, Appah D, Abanum AM, Appah D (2013). Laboratory studies of chemicals for sand consolidation (Scon) in the Niger Delta fields. SPE Nigeria Annual International Conference and Exhibition.

[24] Mishra S, Ojha K (2016). Application of an improvised inorganic–organic chemical mixture to consolidate loose sand formations in oil fields. J. Petrol. Sci. Eng..

[25] Mishra S, Ojha K (2016). Nanoparticle induced chemical system for consolidating loosely bound sand formations in oil fields. J. Petrol. Sci. Eng..

[26] Liu J (2018). Evaluation of strength properties of sand modified with organic polymers. Polymers.

[27] Marandi SZ, Salehi MB, Moghadam AM (2018). Sand control: Experimental performance of polyacrylamide hydrogels. J. Petrol. Sci. Eng..

[28] Sugihardjo S (2020). Evaluation of chemical for sand consolidation in laboratory scale. Sci. Contrib. Oil Gas.

[29] Tabbakhzadeh MN (2020). Experimental study of chemical sand consolidation using epoxy and furan resins for oil wells: Experimental design models. Int. J. Rock Mech. Min. Sci..

[30] Alanqari K, Alanqari K (2019). Innovative epoxy resin formulation. International Petroleum Technology Conference.

[31] Nejati H (2023). Synthesis and optimization of a Novel epoxy-based nanofluid for sand consolidation in oil wells. Geoenergy Sci. Eng..

[32] Tan YH (2017). Application of RSM and Taguchi methods for optimizing the transesterification of waste cooking oil catalyzed by solid ostrich and chicken-eggshell derived CaO. Renew. Energy.

[33] Khalifa AE, Lawal DU (2016). Application of response surface and Taguchi optimization techniques to air gap membrane distillation for water desalination—A comparative study. Desalin. Water Treat..

[34] Chen W-H (2021). Optimization and analysis of syngas production from methane and CO_2_ via Taguchi approach, response surface methodology (RSM) and analysis of variance (ANOVA). Fuel.

[35] Dargi M, Khamehchi E, Mahdavi Kalatehno J (2023). Optimizing acidizing design and effectiveness assessment with machine learning for predicting post-acidizing permeability. Sci. Rep..

[36] Kalatehno JM (2023). A novel approach to determining appropriate additive concentrations for stimulation of gas carbonate reservoirs. Results Eng..

[37] Kalatehno JM, Khamehchi E (2021). A novel packer fluid for completing HP/HT oil and gas wells. J. Petrol. Sci. Eng..

[38] Yousefmarzi F (2024). Machine learning approaches for estimating interfacial tension between oil/gas and oil/water systems: A performance analysis. Sci. Rep..

[39] Khamehchi E, Tabibzadeh S, Alizadeh A (2016). Rheological properties of Aphron based drilling fluids. Petrol. Explor. Dev..

[40] Alizadeh A, Khamehchi E (2019). Experimental investigation of the oil based Aphron drilling fluid for determining the most stable fluid formulation. J. Petrol. Sci. Eng..

[41] Shang X (2019). A novel chemical-consolidation sand control composition: Foam amino resin system. e-Polymers.

[42] Dees JM, Begnaud WJ, Sahr NL (1992). Sand Control with Resin and Explosive.

[43] Fader P, Fader P (1992). New low-cost resin system for sand and water control. SPE Western Regional Meeting.

[44] Parlar M, Parlar M (1998). New chemistry and improved placement practices enhance resin consolidation: Case histories from the Gulf of Mexico. SPE International Conference and Exhibition on Formation Damage Control.

[45] Cobianco S, Cobianco S (1999). Dirty sand consolidation technology for gas wells. SPE International Conference on Oilfield Chemistry?.

[46] Larsen T, Larsen T (2006). Quasinatural consolidation of poorly consolidated oilfield reservoirs. SPE International Oilfield Scale Conference and Exhibition?.

[47] Chaloupka V, Chaloupka V (2010). Remedial sand consolidation: Case study from Mahakam Delta, Indonesia. SPE International Symposium and Exhibition on Formation Damage Control.

[48] Al-Mulhem AA (2019). Consolidated Material to Equalize Fluid Flow into a Wellbore.

